# Stage-Dependent Anthelmintic Activity of Licarin A Against the Neurotropic Nematode *Angiostrongylus cantonensis*

**DOI:** 10.3390/tropicalmed11100263

**Published:** 2026-09-23

**Authors:** Elias L. Neves, Lucas Fukui-Silva, Thainá R. Teixeira, Davi Luna-Tavares, Felipe S. Sales, João Henrique G. Lago, Nadjar Nitz, Josué de Moraes

**Affiliations:** 1 Research Center on Neglected Diseases, Brazil University, Guarulhos 07023-070, SP, Brazil; eliasluiz1405@gmail.com (E.L.N.); fukui.npdn@gmail.com (L.F.-S.); thainanpdn@gmail.com (T.R.T.); 2 Interdisciplinary Laboratory of Biosciences, Faculty of Medicine, University of Brasília, Brasília 70910-900, DF, Brazil; 3 Research Center on Neglected Diseases, Scientific and Technological Institute, Brazil University, São Paulo 08230-030, SP, Brazil; davilunatav@gmail.com; 4 Center for Natural and Human Sciences, Federal University of ABC, São Paulo 09210-180, SP, Brazil; felipe.sales@aluno.ufabc.edu.br

**Keywords:** ADMET, anthelmintic drug discovery, natural products, neolignan, neuroangiostrongyliasis, phenotypic screening, rat lungworm, stage-dependent activity

## Abstract

*Angiostrongylus cantonensis* is the leading cause of eosinophilic meningitis worldwide, but therapeutic options remain limited. This study investigated the anthelmintic activity and preliminary selectivity of licarin A, a neolignan isolated from *Nectandra oppositifolia*. The compound was evaluated *in vitro* against first-stage (L1) larvae, infective third-stage (L3) larvae, and adult worms, using albendazole as the reference drug. Cytotoxicity was assessed in mammalian kidney epithelial, keratinocyte, and neuronal cell models, while whole-organism toxicity was examined in *Caenorhabditis elegans*. Physicochemical, pharmacokinetic, drug-likeness, and toxicological properties were also predicted computationally. Licarin A was active against L1 and L3 larvae, with EC_50_ values of 16.8 and 36.4 µM, respectively. Its activity was comparable to albendazole against L1 but lower against L3, and no relevant effect was observed against adult worms (EC_50_ > 100 µM). No detectable cytotoxicity or lethality was observed in the mammalian cell lines or *C. elegans* at concentrations up to 500 µM. Computational analyses indicated favorable drug-likeness, high gastrointestinal absorption, blood–brain barrier permeation, lack of recognition as a P-glycoprotein substrate, and generally low predicted toxicity. These findings identify licarin A as a selective, stage-dependent anthelmintic hit and provide a foundation for optimization of its scaffold against parasitic nematodes.

## 1. Introduction

*Angiostrongylus cantonensis*, commonly known as the rat lungworm, is a neurotropic nematode and the leading cause of eosinophilic meningitis worldwide [1,2]. Although historically associated with Southeast Asia and the Pacific region, the parasite has expanded geographically and is increasingly recognized as an emerging zoonotic threat [3,4]. Humans become accidental hosts after ingesting infective third-stage larvae (L3) in mollusks, paratenic hosts, or contaminated vegetables. Following infection, the larvae migrate to the central nervous system, where their presence and subsequent death can trigger intense inflammation and neurological manifestations [5]. Because the parasite does not complete its life cycle in humans, disease is primarily associated with migrating larval stages [6].

The management of neuroangiostrongyliasis remains challenging. Albendazole is frequently administered, often in combination with corticosteroids, but therapeutic outcomes may vary according to the timing of treatment, parasite stage, and host inflammatory response [7,8]. These limitations emphasize the need for new anthelmintic agents capable of acting against tissue-migrating larvae and, ideally, reaching sites of infection within the central nervous system [9,10]. Whole-organism phenotypic assays using *A. cantonensis* provide a useful platform for identifying such compounds [11,12,13]. First-stage larvae (L1) offer an accessible model for initial screening [14], whereas L3 represent the infective stage directly associated with human disease [12,13]. Evaluation across additional developmental stages can further define the spectrum and stage specificity of candidate compounds [15]. In this context, natural products constitute an important source of structurally diverse molecules for anthelmintic drug discovery [16,17].

Licarin A is a naturally occurring dihydrobenzofuran neolignan reported in several plant species, including *Nectandra oppositifolia* (Lauraceae) [18]. The compound has attracted pharmacological interest because of its broad range of biological properties, including antiparasitic activity [19,20,21]. Previous studies demonstrated that licarin A affects the viability and motility of adult *Schistosoma mansoni in vitro* [21,22]. Moreover, oral administration to infected mice partially reduced worm and egg burdens and ameliorated infection-associated hepatosplenomegaly [22]. These findings established the anti-helminthic potential of licarin A; however, its activity against parasitic nematodes has not been investigated.

To address this gap, licarin A isolated from *N. oppositifolia* was evaluated against L1 larvae, infective L3 larvae, and adult worms of *A. cantonensis*, using albendazole as the reference anthelmintic. Its preliminary safety and selectivity were assessed in three mammalian cell lines and in the free-living nematode *Caenorhabditis elegans*. In addition, physicochemical, pharmacokinetic, drug-likeness, and toxicological properties were investigated using *in silico* approaches. This integrated strategy aimed to define the stage-specific activity of licarin A and assess its potential as a natural-product scaffold for anthelmintic drug discovery.

## 2. Materials and Methods

### 2.1. General Experimental Procedures

NMR spectra were recorded on a Bruker Ascend Evo 600 spectrometer (Bruker, Billerica, MA, USA), respectively, operating at 600 and 150 MHz for ^1^H and ^13^C nuclei. Spectra were acquired in CDCl_3_ (Aldrich, St. Louis, MO, USA), with tetramethyl silane (TMS; Aldrich) used as the internal standard. High-resolution electrospray ionization mass spectrometry (HR-ESI-MS) data were obtained using a Bruker Daltonics MicroTOF QII spectrometer (Bruker, Billerica, MA, USA). Optical rotation values were measured in a JASCO P-2000 polarimeter (Jasco Corporation, Tokyo, Japan), with Na filter (λ = 589 nm). Silica gel 60 (63–210 mesh Merck, Darmstadt, Germany) and silica gel 60 PF254 (Merck, Darmstadt, Germany) were used for column chromatography and analytical thin-layer chromatography (TLC; 0.25 mm thickness), respectively.

### 2.2. Plant Material

Aerial parts of *N. oppositifolia*, in the vegetative state, were collected in November 2025 at Parque Estadual Alberto Loefgren (23°27′43.97″ S, 46°37′59.43″ W), São Paulo, São Paulo State, Brazil, from the same specimen previously investigated by our research group [22]. The plant material was identified by Dr. Guilherme M. Antar and a voucher specimen was compared with that deposited in the Herbarium of Institute of Biosciences, University of São Paulo, SP, Brazil, under code SPF 225339.

### 2.3. Extraction and Isolation of Licarin A

Dried aerial parts of *N. oppositifolia* (400 g) were powdered and extracted by maceration with *n*-hexane (6 × 1 L) at room temperature. After removal of the solvent under reduced pressure, 24 g of the *n*-hexane extract were obtained. To isolate licarin A, a portion of the extract (10 g) was subjected to silica gel column chromatography, using *n*-hexane with increasing proportions of EtOAc as the mobile phase. A total of 48 fractions (50 mL each) were collected and pooled into six groups (A–F) based on TLC analysis. A portion of group E (1.6 g), eluted with *n*-hexane:EtOAc (8:2), was further purified by silica gel column chromatography using *n*-hexane:EtOAc mixtures (8:2, 7:3, 1:1, and 2:8), affording 544 mg of licarin A (99% purity as determined by HPLC).

*Licarin A*. [α]_D_^24^ −63 (*c* 0.11, MeOH). ^1^H NMR (CDCl_3_), δ/ppm: 6.97 (d, *J* = 1.4 Hz, H-2), 6.92 (dd, *J* = 7.8 and 1.4 Hz, H-6), 6.91 (d, *J* = 7.8 Hz, H-5), 6.79 (br s, H-2′), 6.77 (br s, H-6′), 6.36 (dd, *J* = 15.7 and 1.6 Hz, H-7′), 6.11 (dq, *J* = 15.7 and 6.6 Hz, H-8′), 5.63 (s, OH), 5.11 (d, J = 9.4 Hz, H-7), 3.90 (s, OCH_3_), 3.89 (s, OCH_3_), 3.45 (dq, *J* = 9.4 and 6.8 Hz, H-8), 1.87 (dd, *J* = 6.6 and 1.6 Hz, H-9′), 1.38 (d, *J* = 6.8 Hz, H-9). ^13^C NMR (CDCl_3_), δ/ppm: 146.7 (C-3), 146.6 (C-4′), 145.8 (C-4), 144.2 (C-5′), 132.2 (C-1′), 132.1 (C-1), 130.9 (C-7′), 123.5 (C-8′), 120.0 (C-6), 114.1 (C-5), 113.3 (C-2′), 113.2 (C-3′), 109.3 (C-6′), 108.9 (C-2), 93.8 (C-7), 56.1 (OCH_3_), 56.0 (OCH_3_), 45.6 (C-8), 18.4 (C-9′), 17.6 (C-9). HR-ESI-MS *m/z* 325.8384 [M − H]^−^ (calcd for C_20_H_21_O_4_ 325.1439)

### 2.4. Parasite and Animal Maintenance

The life cycle of *A. cantonensis* was maintained at the Research Center on Neglected Diseases (NPDN), Guarulhos University, Brazil, using *Lissachatina fulica* as the intermediate host and Wistar rats (*Rattus norvegicus*) as definitive hosts. Parasite maintenance and experimental infections were conducted under controlled laboratory conditions in accordance with established institutional procedures. The free-living nematode *C. elegans* (Bristol N2 strain) was cultured on nematode growth medium (NGM) agar plates seeded with *Escherichia coli* OP50 as a nutrient source [23].

### 2.5. Anthelmintic Assays with A. cantonensis Larvae (L1 and L3)

First-stage (L1) larvae of *A. cantonensis* were obtained from the feces of experimentally infected Wistar rats using the Rugai sedimentation technique [24], followed by washing in RPMI 1640 medium supplemented with antibiotics, as previously described [25]. Third-stage (L3) larvae were recovered from experimentally infected *L. fulica* specimens by artificial digestion [26], followed by sedimentation and larval recovery, according to a previously established methodology [27]. Approximately 50 larvae were transferred to each well of 96-well culture plates containing RPMI 1640 medium [28]. Licarin A was initially evaluated at a concentration of 100 μM and subsequently tested at lower concentrations through two-fold serial dilutions, resulting in final concentrations of 100, 50, 25, 12.5, and 6.25 μM [13]. Albendazole was included as the reference anthelmintic and phenotypic control, while untreated larvae maintained under the same experimental conditions served as the negative control. All experimental conditions were evaluated in triplicate.

Following exposure to the treatments, the plates were maintained under controlled incubation conditions. Larval motility was assessed by microscopic examination after 1 min and 24 h of exposure, based on the degree of movement, and scored using a previously established scoring system for the phenotypic evaluation of *A. cantonensis* [14]. An anthelmintic effect was determined based on the reduction in larval motility relative to the untreated control group.

### 2.6. Anthelmintic Assay with Adult A. cantonensis

Adult *A. cantonensis* worms were recovered from the lungs of previously infected Wistar rats [29], and subsequently transferred to RPMI 1640 medium supplemented with antibiotics. Approximately one pair of adult worms, consisting of one male and one female, was transferred to each well of 12-well culture plates. Licarin A was initially tested at 100 μM and subsequently evaluated at lower concentrations using serial dilutions [30]. Albendazole was included as the reference anthelmintic and positive control, whereas untreated worms maintained under the same experimental conditions served as the negative control. All experimental conditions were evaluated in triplicate.

Following 24 h of exposure, adult worm motility and viability were assessed by optical microscopy according to previously established procedures for the evaluation of *A. cantonensis* [14]. Anthelmintic activity was determined based on the reduction in worm motility and viability relative to the untreated control group.

### 2.7. Cytotoxicity Assay in Mammalian Cell Lines

HaCaT (human keratinocyte; BCRJ-0341) and SH-SY5Y (human neuroblastoma; BCRJ-0223) cell lines were obtained from the Banco de Células do Rio de Janeiro (BCRJ, Duque de Caxias, RJ, Brazil), whereas Vero cells (African green monkey kidney epithelial cells; ATCC CCL-81) were purchased from the American Type Culture Collection (Manassas, VA, USA). SH-SY5Y cells were cultured in a 1:1 mixture of Dulbecco’s modified Eagle’s medium (DMEM) and Ham’s F12 medium supplemented with 10% fetal bovine serum (FBS). HaCaT and Vero cells were maintained in DMEM supplemented with 10% FBS [13]. All culture media were supplemented with penicillin (100 U/mL) and streptomycin (100 μg/mL). Cells were maintained at 37 °C in a humidified atmosphere containing 5% CO_2_ [31].

For the cytotoxicity assays, cells were seeded in 96-well plates at a density of 2 × 10^3^ cells/well and incubated under standard culture conditions [32]. After cell attachment, cells were exposed to licarin A at an initial concentration of 500 μM, followed by serial dilutions. Doxorubicin (15 μM) was used as the positive control, whereas 0.5% DMSO served as the vehicle control. After 24 h of exposure, cell viability was assessed using the MTT assay [33]. Briefly, MTT solution was added to each well, and the plates were incubated for an additional 4 h. The resulting formazan crystals were dissolved, and absorbance was measured at 595 nm. Cell viability was expressed as a percentage relative to the untreated control wells [34].

### 2.8. Lethal Toxicity Assay in C. elegans

The potential toxic effects of licarin A were evaluated using fourth-stage larvae (L4) of *C. elegans*. Synchronized L4 larvae were transferred to 96-well plates containing culture medium at an approximate density of 60 larvae per well [27]. Licarin A was initially evaluated at 500 μM and subsequently tested at lower concentrations through two-fold serial dilutions, resulting in final concentrations of 500, 250, 125, and 62.5 μM [28]. Ivermectin (5 μM) was used as the positive control for lethality, whereas untreated larvae maintained in culture medium served as the negative control. All experimental conditions were evaluated in triplicate.

After 24 h of exposure, larval viability was assessed by direct microscopic observation of spontaneous locomotion and locomotor responses following mechanical stimulation. Larvae exhibiting no detectable movement, including after direct mechanical stimulation, were classified as nonviable. The negative control exhibited no detectable lethality, whereas ivermectin produced complete larval lethality under the experimental conditions. Licarin A was considered toxic at concentrations resulting in >50% lethality of the exposed population, whereas concentrations associated with ≤50% lethality were classified as non-toxic [35].

### 2.9. In Silico ADME Analysis

*In silico* analysis of the physicochemical and pharmacokinetic properties of Licarin A was performed using the SwissADME web-based platform [36]. Parameters including molecular weight, lipophilicity, polarity, solubility, gastrointestinal absorption, and drug-likeness were evaluated using the platform’s default settings. The results were used for a preliminary assessment of the ADME profile of Licarin A and its characteristics related to its potential as a drug development candidate.

### 2.10. In Silico Toxicity Prediction

Acute oral toxicity (LD_50_), GHS classification, and organ-specific toxicity end points were predicted using the ProTox-3.0 platform [37].

### 2.11. Statistical Analysis

All experiments were performed in triplicate. Data were analyzed using GraphPad Prism version 8.0 (GraphPad Software, San Diego, CA, USA). Concentration–response curves were fitted by nonlinear regression, and EC_50_ values were calculated with corresponding 95% confidence intervals (95% CI). For comparisons among experimental groups, one-way analysis of variance (ANOVA) followed by Tukey’s multiple-comparison test was used [14].

### 2.12. Ethical Statement

All animal procedures were approved by the Institutional Animal Care and Use Committee of Guarulhos University (protocol no. 064/24).

## 3. Results

### 3.1. Isolation and Identification of Licarin A

Licarin A was isolated from the aerial parts of *N. oppositifolia* through several chromatographic steps. Its structure (Figure 1) was established by NMR, HR-ESI-MS and optical rotation followed by comparison with previously reported data [22].

### 3.2. Licarin A Exhibits Stage-Dependent Activity Against A. cantonensis

The anthelmintic activity of licarin A was evaluated against first-stage larvae (L1), infective third-stage larvae (L3), and adult worms of *A. cantonensis*. Licarin A showed concentration-dependent activity against both larval stages, with EC_50_ values of 16.8 µM against L1 and 36.4 µM against L3 (Table 1 and Figure 2). Thus, the compound was approximately 2.2-fold more potent against L1 than against L3.

Against L1, the activity of licarin A did not differ significantly from that of albendazole, which exhibited an EC_50_ of 15.7 µM. In contrast, licarin A was significantly less potent than albendazole against L3 (*p* < 0.05), for which the reference drug showed an EC_50_ of 16.3 µM.

Licarin A did not reach 50% activity against adult worms at concentrations up to 100 µM, whereas albendazole was active against this stage, with an EC_50_ of 7.2 µM. These findings demonstrate a stage-dependent activity profile, with licarin A acting preferentially against the larval stages of *A. cantonensis*.

### 3.3. Licarin A Shows Low Toxicity Toward Mammalian Cells and C. elegans

The cytotoxicity of licarin A was evaluated in Vero, SH-SY5Y, and HaCaT cells. The compound did not reduce cell viability by 50% at the highest concentration evaluated, resulting in CC_50_ values above 500 µM in all three cell lines (Table 2). Based on the lowest CC_50_ value, licarin A exhibited selectivity indices greater than 29 against L1 and greater than 13 against L3. A selectivity index could not be determined for adult worms because an EC_50_ value was not established within the tested concentration range (Figure 3a).

The toxicity of licarin A was further examined in *C. elegans* (Figure 3b). The compound did not reach 50% lethality at concentrations up to 500 µM, resulting in an LC_50_ above 500 µM. In contrast, albendazole exhibited an LC_50_ of 15.9 µM under the same experimental conditions. Collectively, these results indicate that licarin A is active against the larval stages of *A. cantonensis* at concentrations substantially below those associated with toxicity in mammalian cells or *C. elegans*.

### 3.4. Licarin A Displays Favorable Predicted Drug-likeness and a Generally Low Toxicity Profile

The predicted physicochemical profile of licarin A was broadly compatible with oral drug-likeness, combining moderate molecular size and polarity, limited molecular flexibility, and favorable lipophilicity. Its overall properties were positioned within the desirable ranges of the bioavailability radar. Licarin A was also predicted to exhibit high gastrointestinal absorption, permeate the blood–brain barrier, and not act as a P-glycoprotein substrate (Figure 4a).

Licarin A fulfilled the Lipinski, Ghose, Veber, Egan, and Muegge drug-likeness criteria and showed a predicted bioavailability score of 0.55. No PAINS or Brenk structural alerts were identified (Figure 4b).

The predicted oral LD_50_ was 4900 mg/kg, corresponding to toxicity class 5 and indicating relatively low acute oral toxicity (Figure 5). Organ-specific models classified licarin A as inactive for hepatotoxicity, neurotoxicity, cardiotoxicity, and respiratory toxicity [37]. A potential nephrotoxicity signal was identified, although its predicted probability was marginal (0.52), indicating low confidence in this classification (Figure 6). Overall, these computational findings support a generally favorable drug-likeness profile while identifying solubility and potential nephrotoxicity as properties requiring experimental investigation.

## 4. Discussion

The present study provides the first evidence of the anthelmintic activity of licarin A against *A. cantonensis* and reveals a stage-dependent profile. Licarin A was active against both larval stages, with greater potency against L1 than against infective L3 larvae, but showed no relevant activity against adult worms at the concentrations evaluated. Its activity against L1 was comparable to that of albendazole, whereas lower potency was observed against L3. This larval activity, combined with low toxicity in mammalian cells and *C. elegans*, resulted in a favorable preliminary selectivity profile. Together with its predicted pharmacological properties, these findings identify licarin A as a selective natural-product scaffold for further anthelmintic investigation.

Comparison with natural products evaluated using the same standardized experimental platform helps position licarin A within the *A. cantonensis* drug-discovery landscape. Against L1 larvae, cubebin [27], spathulenol [25], sakuranetin [15], and piplartine [28] exhibited EC_50_ values of 4.7, 7.6, 8.1, and 8.3 µM, respectively, while phendione and its metal complexes showed values ranging from 6.4 to 25.6 µM [38]. Against L3 larvae, its EC_50_ of 36.4 µM was higher than those reported for piplartine [28] and cubebin [27] but lower than that of sakuranetin [15], which showed a pronounced reduction in potency against this stage. Thus, although licarin A was not among the most potent compounds identified in this model, its activity was comparable to albendazole against L1 and its measurable effect against the infective L3 stage support its classification as a bioactive phenotypic hit candidate. Within the Damage Response Framework [39], this stage-dependent phenotypic activity provides an initial basis for considering licarin A as a phenotypic hit candidate, while further target identification and validation will be required to define its molecular mechanism.

The progressive reduction in activity from L1 to L3, followed by the lack of relevant activity against adults, indicates that developmental stage is an important determinant of susceptibility to licarin A. This profile differs from that recently reported for andrographolide, which retained activity across L1, L3, and adult *A. cantonensis* [30]. The highly consistent activity of albendazole across the two studies supports the reproducibility of the experimental platform and indicates that the lower susceptibility of adult worms to licarin A represents a compound-specific biological response. Developmental differences in cuticle composition, compound uptake, xenobiotic metabolism, or molecular target expression could contribute to this pattern [40,41]. Although adult worms are not the primary therapeutic target in human neuroangiostrongyliasis, their inclusion helps delineate the stage-specific spectrum of the compound.

An important finding of the present study was the favorable experimental selectivity of licarin A. The compound showed no detectable cytotoxicity in mammalian cell lines representing kidney epithelial, keratinocyte, and neuronal models, resulting in favorable selectivity toward L1 and L3 larvae. In addition, licarin A caused no detectable lethality in *C. elegans*, whereas albendazole exhibited a low LC_50_. This marked difference suggests that the activity of licarin A against *A. cantonensis* is unlikely to result from nonspecific toxicity toward nematodes. Nevertheless, *C. elegans* should be considered a complementary whole-organism toxicity model rather than a direct surrogate for parasite-specific selectivity [42,43], and differences in compound uptake, metabolism, or target biology may account for the distinct responses of the two nematodes.

The experimental selectivity was complemented by a favorable predicted pharmacological and toxicological profile, including compliance with major drug-likeness criteria and the absence of structural alerts. Licarin A was predicted to exhibit high gastrointestinal absorption, permeate the blood–brain barrier, and not act as a P-glycoprotein substrate. These properties are potentially relevant to neuroangiostrongyliasis, in which infective larvae migrate to the central nervous system [28,44]. The predicted acute and organ-specific toxicity was also generally low, although a low-confidence nephrotoxicity signal was identified. These computational findings should be regarded as preliminary indicators because they do not establish systemic exposure, brain penetration, or safety *in vivo*.

The principal limitation of this study is that the activity of licarin A against *A. cantonensis* was demonstrated exclusively under *in vitro* conditions. No mechanistic, pharmacokinetic, tissue-distribution, or *in vivo* efficacy investigations were conducted; therefore, it remains uncertain whether biologically active concentrations can be achieved at the sites of infection, particularly within the central nervous system. Future studies should consequently evaluate systemic and brain exposure, tolerability, and the efficacy of licarin A in experimental models of angiostrongyliasis. In addition, mechanistic investigations are warranted to elucidate the molecular determinants underlying its activity, as well as to identify and validate potential biological targets, thereby contributing to a better understanding of the mechanisms associated with parasite susceptibility. Finally, the present findings establish a biological benchmark for structure–activity relationship studies and optimization of the licarin A scaffold, particularly with the aim of enhancing its activity against L3 while preserving its favorable selectivity profile.

## 5. Conclusions

Licarin A, a neolignan isolated from *N. oppositifolia*, exhibited selective and stage-dependent activity against *A. cantonensis*, with potency comparable to albendazole against L1 larvae, moderate activity against infective L3 larvae, and no relevant effect against adult worms. Its low toxicity in mammalian cells and *C. elegans*, together with its favorable predicted drug-likeness and pharmacokinetic profile, supports its classification as a selective anthelmintic hit. These findings establish the activity of licarin A against a parasitic nematode and provide a biological foundation for structure–activity relationship studies, mechanistic investigation, and evaluation in experimental angiostrongyliasis.

## Figures and Tables

**Figure 1:**
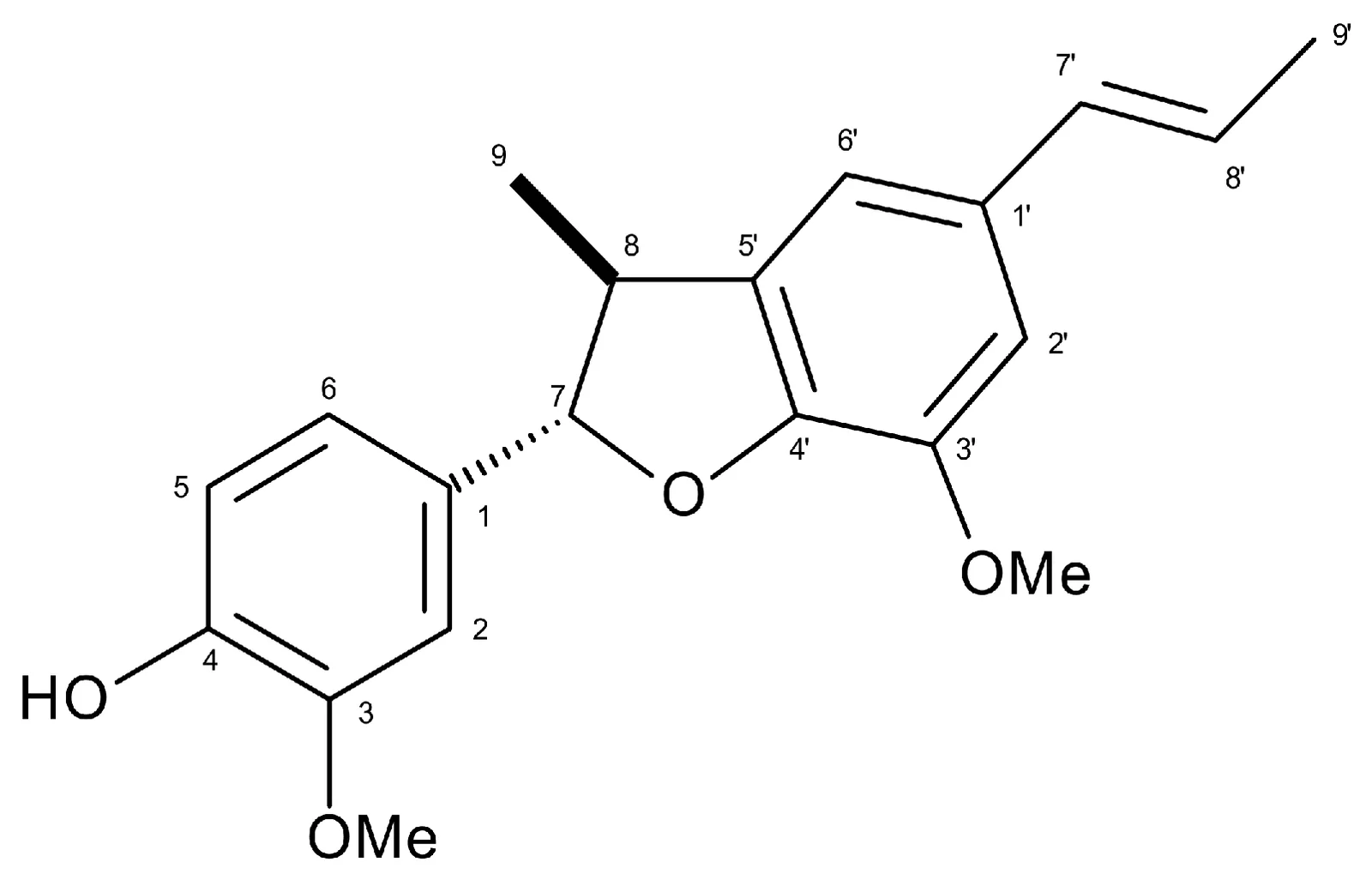
Chemical structure of licarin A.

**Figure 2:**
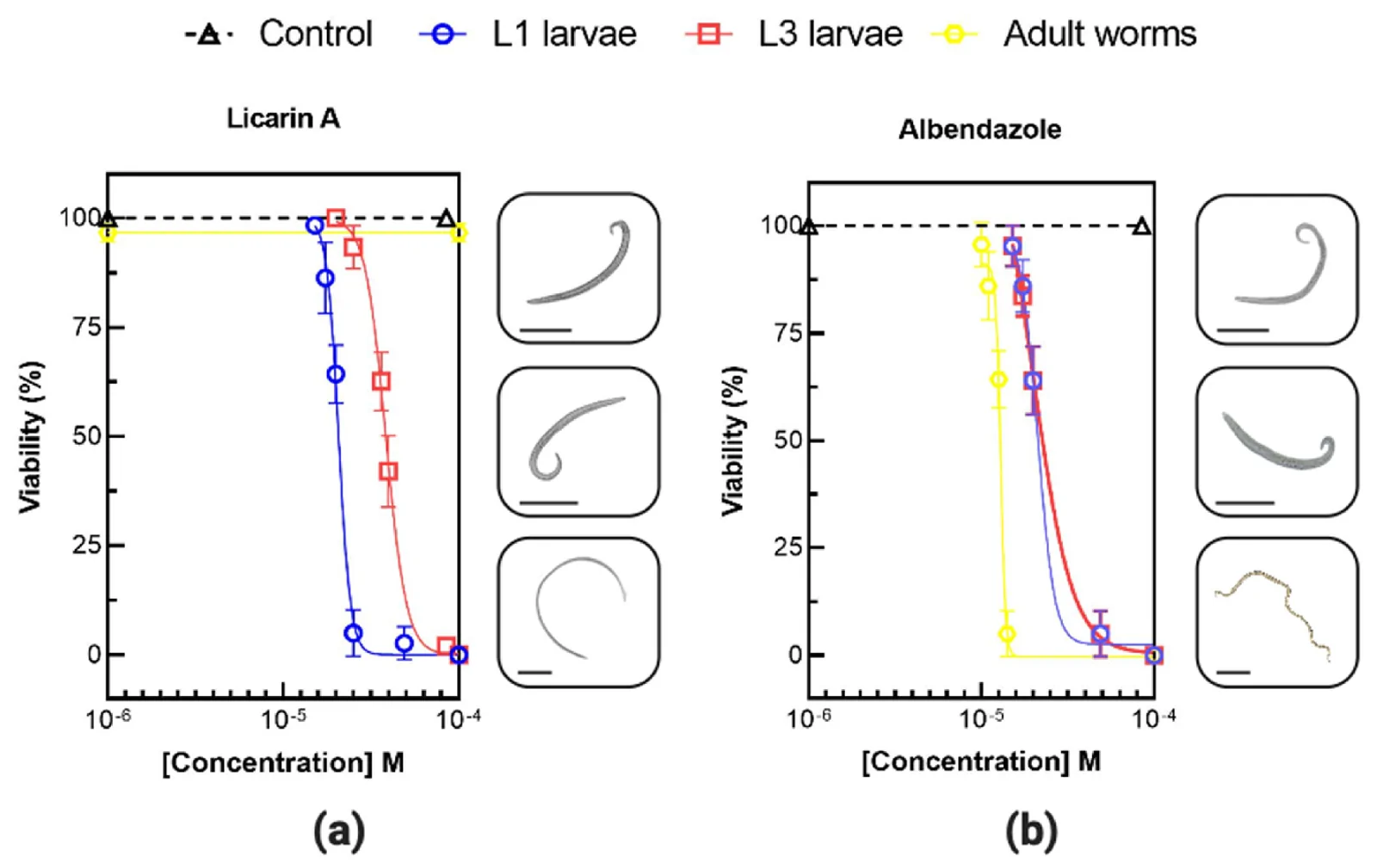
Concentration–response activity of licarin A and albendazole against different developmental stages of *A. cantonensis*. Viability of first-stage larvae (L1; blue), infective third-stage larvae (L3; red), and adult worms (yellow) after 24 h of exposure to (**a**) licarin A or (**b**) albendazole. Black dashed lines and open triangles represent the negative controls. Solid lines represent nonlinear regression fits used to estimate EC_50_ values. Data are presented as the mean ± SD of three independent experiments. Representative images of L1 larvae, L3 larvae, and adult worms are identified directly in the figure. Scale bars: L1, 35 µm; L3, 35 µm; adult worms, 200 µm.

**Figure 3:**
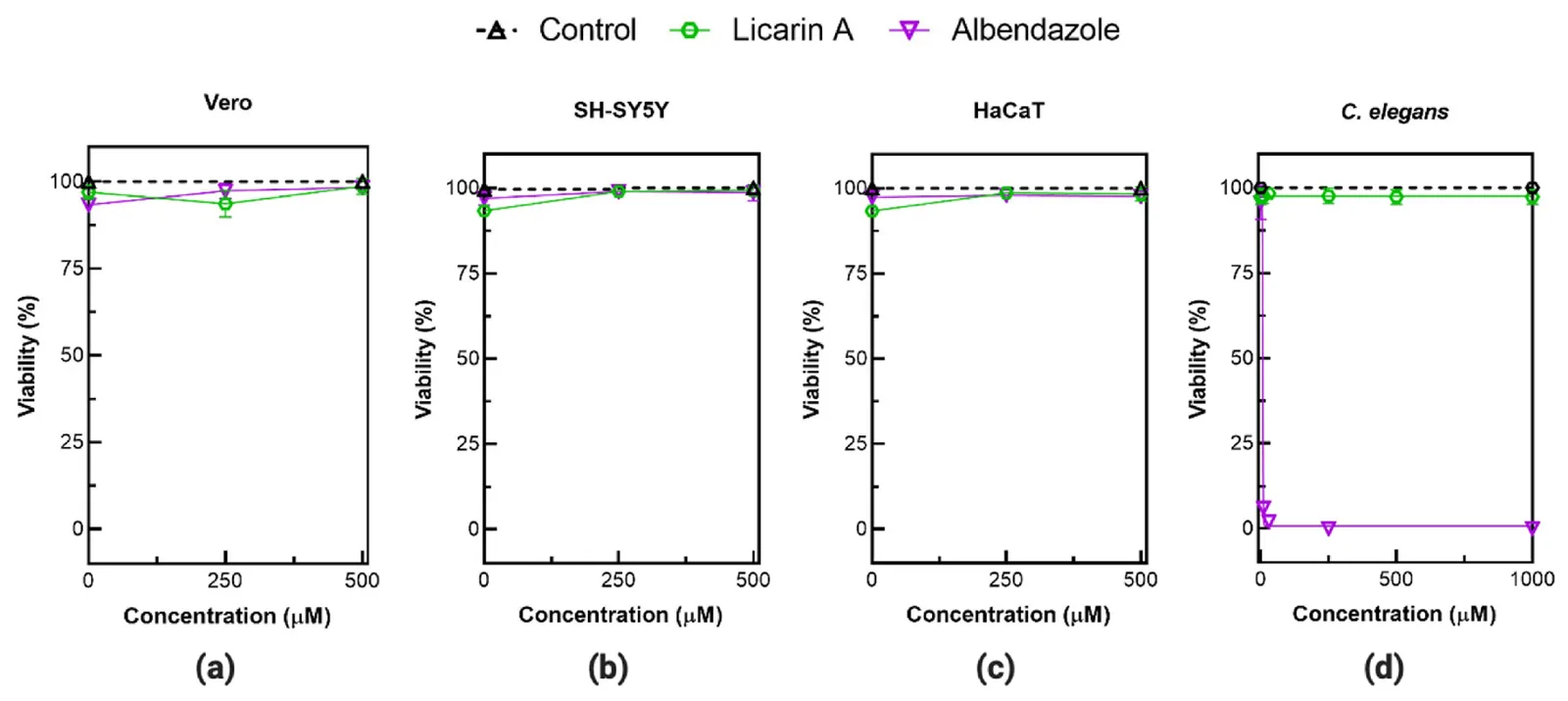
Effects of licarin A and albendazole on the viability of mammalian cells and *C. elegans*. Concentration–viability profiles were determined in (**a**) kidney epithelial cells (Vero), (**b**) neuronal cells (SH-SY5Y), (**c**) keratinocytes (HaCaT), and (**d**) *C. elegans*. Licarin A and albendazole are represented by green circles and purple inverted triangles, respectively. Black triangles and dashed lines represent the respective controls. Data are presented as the mean ± SD of three independent experiments.

**Figure 4:**
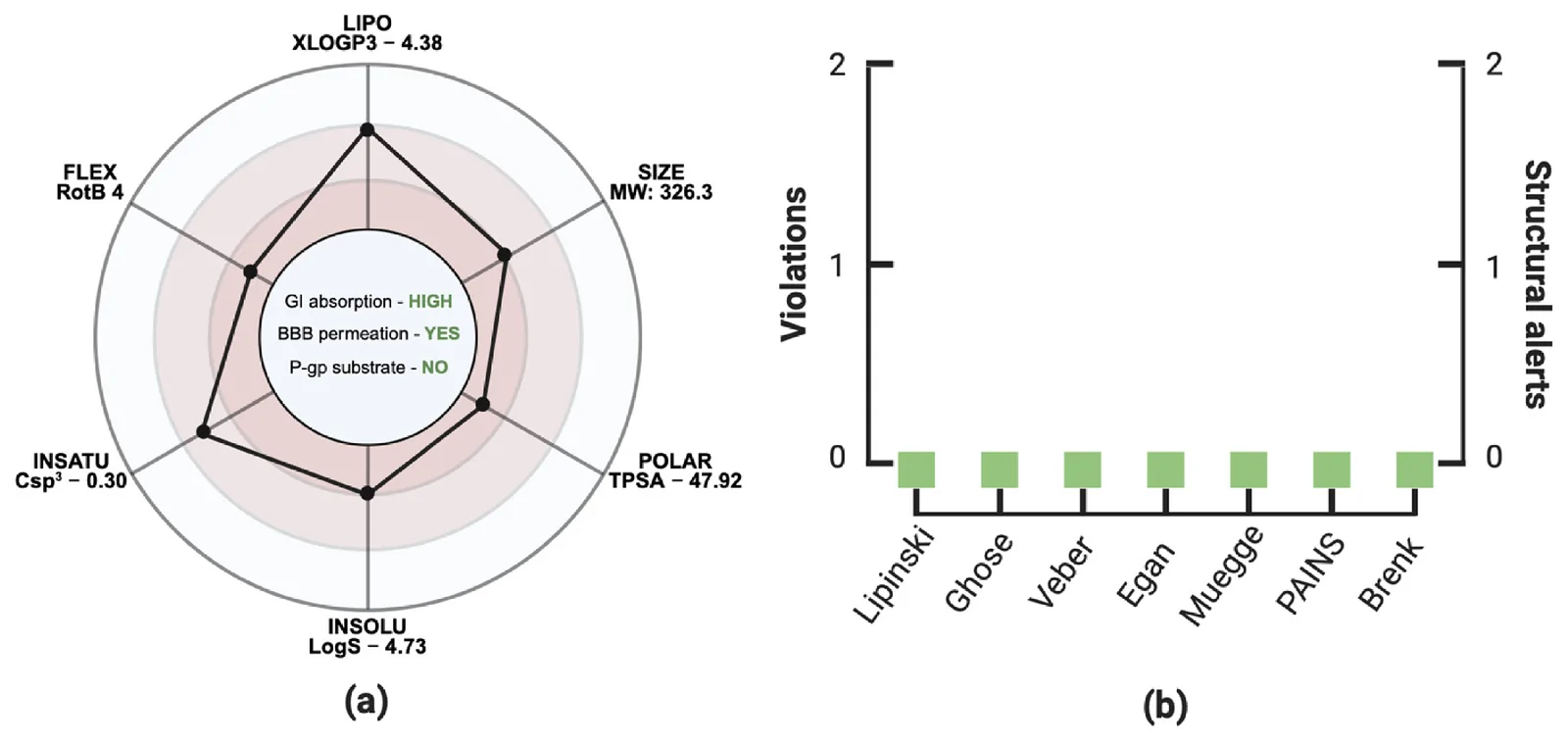
Predicted physicochemical, pharmacokinetic, and drug-likeness profile of licarin A. (**a**) Circular representation of the bioavailability profile. The shaded annular region indicates the optimal range for each physicochemical property, and the values and radial positions of licarin A were normalized to the corresponding property-specific intervals. The central area summarizes the predicted high gastrointestinal absorption, blood–brain barrier permeation, and absence of recognition as a P-glycoprotein substrate. LIPO, lipophilicity; SIZE, molecular size; POLAR, polarity; INSOLU, insolubility; INSATU, unsaturation; FLEX, flexibility. (**b**) Numbers of violations of the Lipinski, Ghose, Veber, Egan, and Muegge drug-likeness criteria and of PAINS and Brenk structural alerts. Licarin A showed no violations or structural alerts.

**Figure 5:**
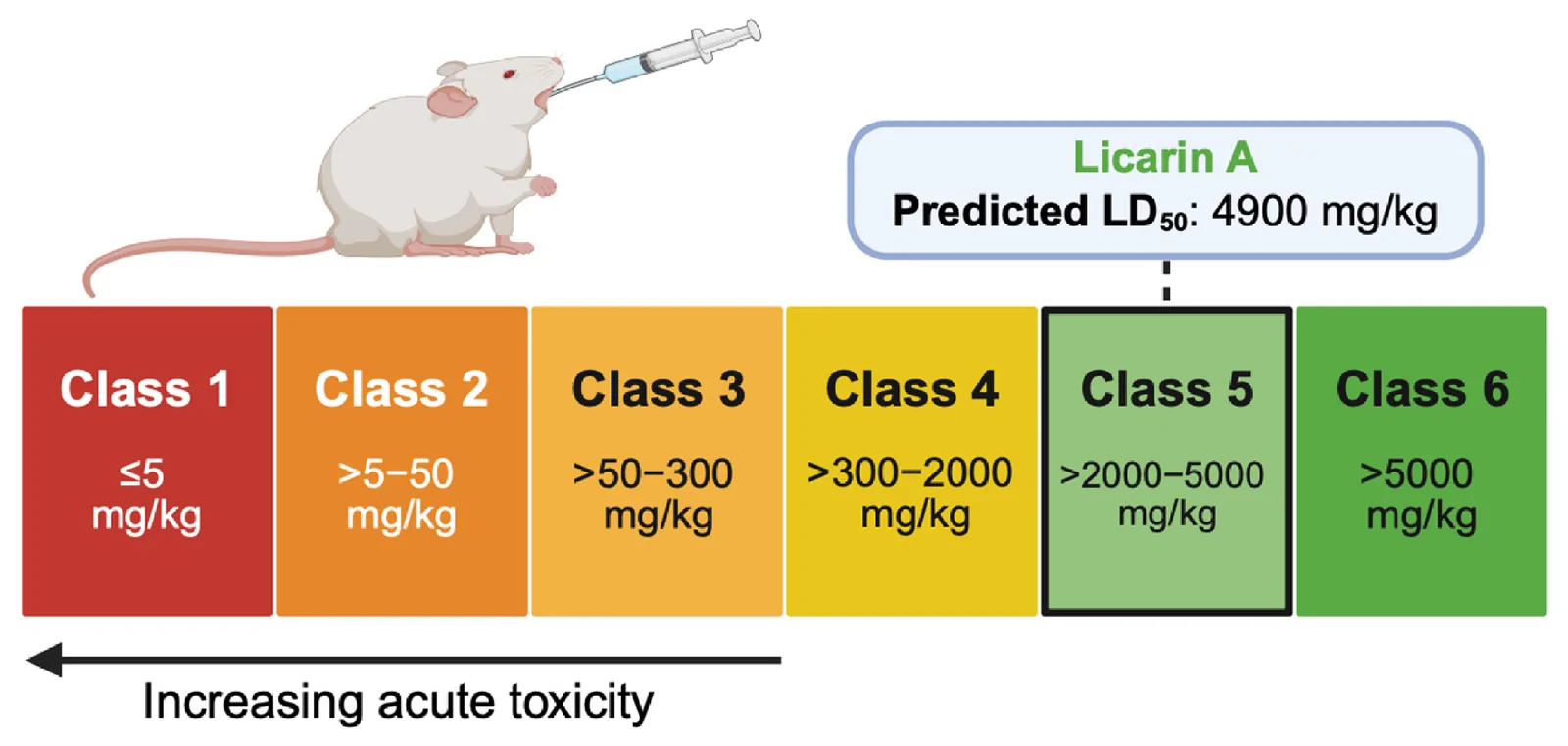
Predicted acute oral toxicity of licarin A. The compound showed a predicted median lethal dose (LD_50_) of 4900 mg/kg in rats and was assigned to toxicity class 5 (>2000–5000 mg/kg), consistent with relatively low predicted acute oral toxicity. The arrow indicates increasing toxicity toward class 1.

**Figure 6:**
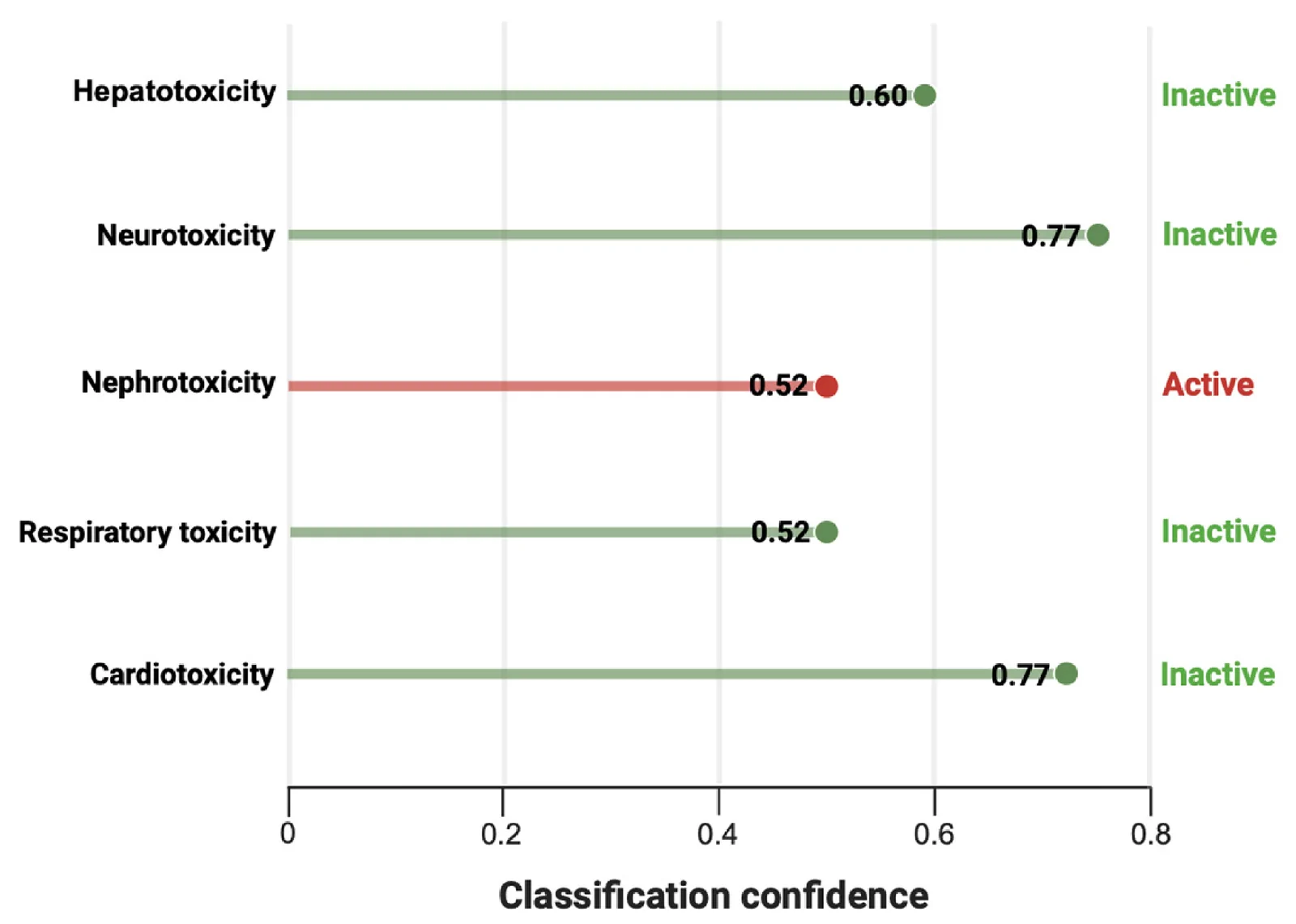
Predicted organ-specific toxicity profile of licarin A. Hepatotoxicity, neurotoxicity, respiratory toxicity, and cardiotoxicity were predicted as inactive, whereas nephrotoxicity was classified as active. Numbers adjacent to the markers indicate the confidence scores of the respective classifications. Green and red denote inactive and active predictions, respectively.

**Table 1:** *In vitro* anthelmintic activity of licarin A and albendazole against different developmental stages of *A. cantonensis*.

Compound	*A. cantonensis* EC_50_ (µM)		
	L1	L3	Adult
Licarin A	16.8 [13.5–20.1]	36.4 [32.9–40.1] *	>100
Albendazole	15.7 [13.3–18.1]	16.3 [14.4–18.5]	7.2 [5.9–8.5]

EC_50_ represents the concentration required to reduce parasite viability by 50% after 24 h of exposure. Values are presented in µM, with 95% confidence intervals shown in brackets. Albendazole was used as the reference anthelmintic. * *p* < 0.05 compared with albendazole within the same developmental stage. The value > 100 indicates that the EC_50_ was not reached at the highest concentration tested.

**Table 2:** Mammalian cell cytotoxicity, *C. elegans* lethality, and selectivity of licarin A and albendazole.

Compound	Mammalian Cells CC_50_ (µM)			Selectivity Index			*C. elegans* LC_50_ (µM)
	Vero	SH-SY5Y	HaCaT	L1	L3	Adults	
Licarin A	>500	>500	>500	>29	>13	ND	>500
Albendazole	>500	>500	>500	>31	>30	>69	15.9 [13.9–19.9]

Cytotoxicity was evaluated in Vero kidney epithelial cells, SH-SY5Y neuronal cells, and HaCaT keratinocytes. CC_50_ represents the concentration that reduced mammalian cell viability by 50%, and LC_50_ represents the concentration that caused 50% lethality in *C. elegans*. The selectivity index (SI) was calculated as CC_50_/EC_50_ for each developmental stage of *A. cantonensis*. Because the CC_50_ was not reached at the highest concentration tested, the corresponding SI values represent lower-bound estimates. Values are expressed in µM, with 95% confidence intervals shown in brackets when calculable. ND not determined.

## Data Availability

The data supporting the findings of this study are available within the article. Further information is available from the corresponding author upon reasonable request.

## Abbreviations

The following abbreviations are used in this manuscript:

ADME	Absorption, Distribution, Metabolism, and Excretion
ANOVA	Analysis of Variance
ATCC	American Type Culture Collection
BCRJ	Rio de Janeiro Cell Bank
CC_50_	50% Cytotoxic Concentration
CDCl_3_	Deuterated Chloroform
CEUA	Ethics Committee on the Use of Animals
CI	Confidence Interval
CO_2_	Carbon Dioxide
DMEM	Dulbecco’s Modified Eagle Medium
DMSO	Dimethyl Sulfoxide
EC_50_	Half-Maximal Effective Concentration
EtOAc	Ethyl Acetate
FBS	Fetal Bovine Serum
FLEX	Flexibility
GHS	Globally Harmonized System of Classification and Labelling of Chemicals
HaCaT	Human Keratinocyte Cell Line
HPLC	High-Performance Liquid Chromatography
HR-ESI-MS	High-Resolution Electrospray Ionization Mass Spectrometry
INSATU	Unsaturation
INSOLU	Insolubility
L1	First-Stage Larvae
L3	Third-Stage Larvae
L4	Fourth-Stage Larvae
LD_50_	Lethal Dose 50%
LIPO	Lipophilicity
MeOH	Methanol
MTT	3-(4,5-Dimethylthiazol-2-yl)-2,5-Diphenyltetrazolium Bromide
ND	Not Determined
NGM	Nematode Growth Medium
NMR	Nuclear Magnetic Resonance
NPDN	Research Center on Neglected Diseases
PAINS	Pan-Assay Interference Compounds
POLAR	Polarity
RPMI 1640	Roswell Park Memorial Institute 1640 Medium
SD	Standard Deviation
SH-SY5Y	Human Neuroblastoma Cell Line
SI	Selectivity Index
SIZE	Molecular Size
TLC	Thin-Layer Chromatography
TMS	Tetramethylsilane
Vero	African Green Monkey Kidney Epithelial Cell Line
